# Mice lacking WRB reveal differential biogenesis requirements of tail-anchored proteins *in vivo*

**DOI:** 10.1038/srep39464

**Published:** 2016-12-21

**Authors:** Jhon Rivera-Monroy, Lena Musiol, Kirsten Unthan-Fechner, Ákos Farkas, Anne Clancy, Javier Coy-Vergara, Uri Weill, Sarah Gockel, Shuh-Yow Lin, David P. Corey, Tobias Kohl, Philipp Ströbel, Maya Schuldiner, Blanche Schwappach, Fabio Vilardi

**Affiliations:** 1 Department of Molecular Biology, Universitätsmedizin Göttingen, D-37073 Göttingen, Germany; 2 Department of Molecular Genetics, Weizmann Institute of Science, Rehovot 7610001, Israel; 3 Department of Cardiology & Pulmonology, Universitätsmedizin Göttingen, D-37075 Göttingen, Germany; 4 Howard Hughes Medical Institute and Department of Neurobiology, Harvard Medical School, Boston, MA, USA; 5 Institute of Pathology, Universitätsmedizin Göttingen, D-37073 Göttingen, Germany; 6 Max-Planck Institute for Biophysical Chemistry, D-37077, Göttingen, Germany

## Abstract

Tail-anchored (TA) proteins are post-translationally inserted into membranes. The TRC40 pathway targets TA proteins to the endoplasmic reticulum via a receptor comprised of WRB and CAML. TRC40 pathway clients have been identified using *in vitro* assays, however, the relevance of the TRC40 pathway *in vivo* remains unknown. We followed the fate of TA proteins in two tissue-specific WRB knockout mouse models and found that their dependence on the TRC40 pathway *in vitro* did not predict their reaction to receptor depletion *in vivo*. The SNARE syntaxin 5 (Stx5) was extremely sensitive to disruption of the TRC40 pathway. Screening yeast TA proteins with mammalian homologues, we show that the particular sensitivity of Stx5 is conserved, possibly due to aggregation propensity of its cytoplasmic domain. We establish that Stx5 is an autophagy target that is inefficiently membrane-targeted by alternative pathways. Our results highlight an intimate relationship between the TRC40 pathway and cellular proteostasis.

The need to target proteins to membranes is a consequence of cellular compartmentalization and thus a fundamental process in all cells. Malleable model organisms such as yeast and reductionist *in vitro* approaches have enabled the elucidation of the basic mechanisms of membrane targeting. Tail-anchored (TA) membrane proteins were recognized^1^ as a type of precursor protein with specific post-translational targeting requirements due to their topology. They represent a subclass of type-II oriented integral membrane proteins with a single transmembrane domain (TMD) at the extreme C-terminus^2^. *In-vitro* studies identified the cytosolic ATPase TRC40 (Transmembrane domain Recognition Complex 40 kDa) as the targeting factor for TA proteins inserted into the membrane of the endoplasmic reticulum (ER) of higher eukaryotes^3^,^4^. A pre-targeting complex including the proteins BAG6, TRC35, and UBL4A receives newly synthesized TA proteins from the ribosome and transfers them to the cytosolic ATPase TRC40^5^. BAG6 also actively promotes degradation of mislocalized proteins by targeting them to the ubiquitin-proteasome system^6^,^7^. TRC40 delivers TA proteins to a receptor formed by WRB (Tryptophan Rich Basic protein)^8^ and CAML (Calcium signal-modulating cyclophilin ligand)^9^, two integral membrane proteins localized at the ER.

A conserved pathway in yeast features Get3, the orthologue of TRC40, and a membrane receptor complex comprised of the proteins Get1 and Get2 as key components^10^. This receptor is thought to work as an insertase for TA proteins at the ER membrane^11^. Many aspects of the molecular mechanism of TRC40/Get3-mediated insertion of TA proteins have been dissected. This dissection gave rise to the current model of TRC40/Get3-dependent targeting that is supported by numerous structural studies of the main components of the pathway in different functional states^12^,^13^,^14^,^15^,^16^.

However, the physiological client spectrum of the TRC40 pathway remains to be identified. In fact, a direct role of the pathway was tested for only a few of the several hundreds theoretically predicted TA proteins^17^ based on *in vitro* membrane integration assays^3^,^4^,^18^. Moreover, yeast Get3 is thought to contribute to the targeting of GPI-anchored proteins^19^ and can work as an ATP-independent chaperone under oxidative stress^20^. The latter observation positions the TRC40/Get pathway at the interface of protein biogenesis and quality control. While both processes make use of fundamental mechanisms that require biochemical and biophysical dissection, their physiological bearing and potential contribution to disease development can only be elucidated in the context of differentiated cell types. Insight into the role of the TRC40 pathway in the context of tissue physiology is currently limited.

Although the GET genes are not essential in yeast^10^, loss of the TRC40^21^ or CAML-encoding^22^ genes result in early embryonic lethality in a murine model. Generation of conditional knockouts restricted to specific cell types allows the identification of relevant functions of components of the TRC40 pathway in tissue development and physiology. A recent study revealed that pancreatic beta cells lacking TRC40 show impaired insulin secretion^23^, whereas CAML is required for thymocyte development^24^, and loss of either receptor subunit in inner ear cells causes deafness^25^,^26^.

The locus of the WRB gene (alternatively named CHD5) was mapped to the congenital heart disease region of human chromosome 21^27^. A recent study showed increased expression of WRB in Down Syndrome (DS) fetal fibroblasts, in accordance with the presence of an extra copy of the WRB allele^28^. However, a potential correlation between overexpression of WRB and the higher incidence of congenital heart disease in DS patients^27^ remains unclear. Knockdown of WRB in medaka fish leads to defects in cardiac development and ocular abnormalities^29^. Similarly, loss of WRB in *Xenopus laevis* has a strong impact on cardiac morphology and secretion of basement membrane matrix^30^. Other studies have identified an essential function of WRB in the formation of synaptic structures in photoreceptors and in inner ear cells^26^,^31^,^32^. Due to the physiological focus on the analysis of the phenotypes, i.e. seeing and hearing deficits, and due to the difficulties in analysing inner hair cells and photoreceptor cells biochemically, these studies provide limited information on the general impact of WRB loss on TA protein biogenesis.

Here, we follow the fate of a subset of TA proteins in two terminally differentiated cell types, cardiomyocytes and hepatocytes, lacking WRB. Our results indicate that the TRC40 pathway is not only active in the targeting and membrane insertion of TA proteins but also part of the proteostatic network with effects on the maintenance and quality control of the cellular proteome.

## Results

### Experimental Strategy

To investigate the role of the TRC40 pathway in tissue physiology, we employed a previously established mouse line with loxP recombination sites introduced into the WRB allele^26^ allowing tissue-specific knockouts upon crossing with mouse lines expressing the Cre recombinase under the control of a promoter of interest. We created tissue-specific knockout models using Cre lines leading to WRB knockout in adult cardiomyocytes or hepatocytes. These target cells were chosen for the potential relevance of WRB function to their physiology and to disease development. The gene encoding WRB was mapped to human chromosome 21 and is associated with congenital heart disease in Down syndrome patients^27^ and severe cardiac developmental disorders in medaka fish^29^ and *Xenopus laevis*^30^. Hepatocytes are active secretory cells and therefore require extensive protein targeting.

### Knockout of WRB in adult cardiomyocytes results in a TRC40 pathway ko

**Figure 1:**
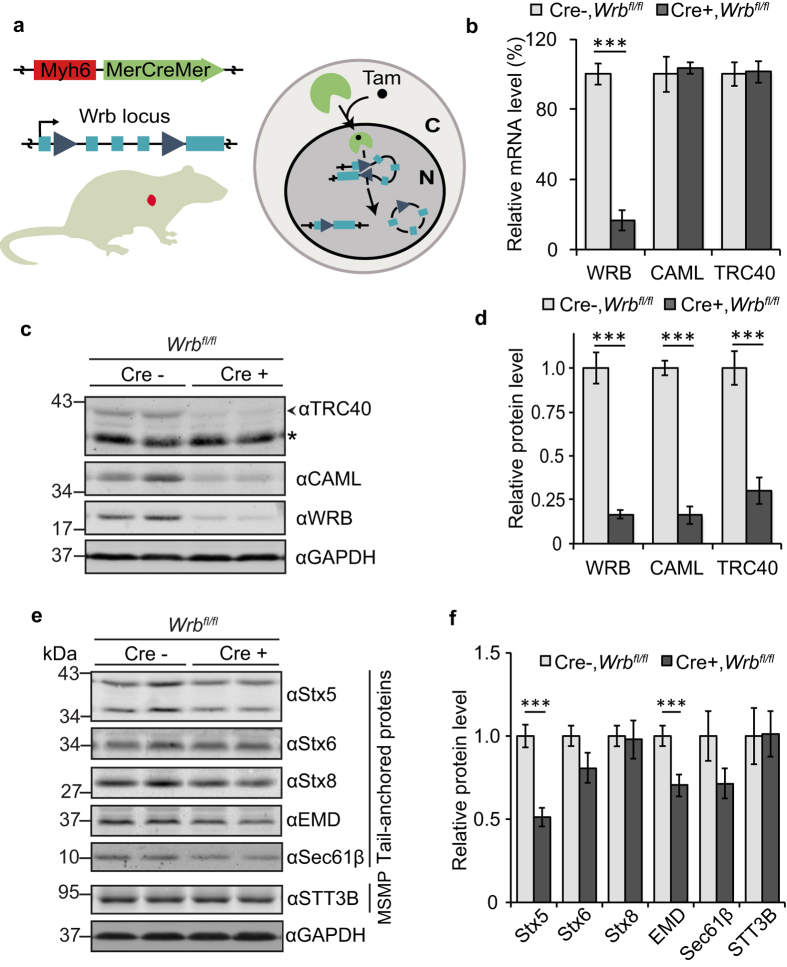
Knockout of WRB in cardiomyocytes results in reduction at steady-state level of TRC40 and CAML as well as Stx5 and emerin (EMD). (**a**) Schematic of the cardiomyocyte-specific tamoxifen inducible knockout of WRB. Administration of tamoxifen triggers nuclear import of MerCreMer recombinase expressed under the control of Myh6 promoter. This leads to recombination of the WRB locus in the WRB^fl/fl^ mouse line. (**b**) mRNA levels of the main TRC40-pathway components relative to GAPDH in eight-week old Myh6-MerCreMer negative and positive littermates two weeks after tamoxifen induction, measured by qPCR. Bars represent average +/− s.e.m. (n = 4, ***p-value < 0.001). (**c**) Cellular lysates from isolated cardiomyocytes were separated by SDS-PAGE and the main TRC40-pathway components were evaluated by western blot using GAPDH as loading control. An asterisk marks a cross-reactive protein. (**d**) Quantification of the blots as in (**c**). Bars represent average +/−s.e.m. (n = 7–16, ***p-value < 0.001). (**e**) Lysates from isolated cardiomyocytes were separated by SDS-PAGE and expression level of different TA proteins and of the multi spanning membrane protein (MSMP) STT3B was analysed by immunoblot using GAPDH as loading control. (**f**) Quantification of the blots as in **e**; bars represent average +/−s.e.m. (n = 6–15, ***p-value < 0.001).

We crossed the mouse line carrying loxP recombination sites flanking exons 2 and 4 of the WRB gene^26^ with a line expressing a Cre recombinase fused to parts of the estrogen receptor (MerCreMer), under control of the cardiomyocyte-specific myosin 6 (Mhy6) promoter^33^. Tamoxifen injection induces nuclear translocation of the recombinase and therefore allowed WRB depletion in a tissue-specific and temporally controlled manner^34^ (Fig. 1a). Two weeks after tamoxifen administration, we isolated cardiomyocytes using the Langendorff perfusion method^35^. Quantitative PCR showed a strong decrease of WRB mRNA in knockout cells whereas transcript levels of TRC40 and CAML were unchanged (Fig. 1b). Western blot analysis confirmed a strong reduction of WRB protein level but also of CAML and TRC40 (Fig. 1c,d). Interdependence of receptor subunit stability was previously reported for the yeast GET receptor (Get1 and Get2)^10^ as well as for WRB and CAML^36^. A recent study has shown that down-regulation of CAML destabilizes WRB mRNA rather than the protein itself^28^. However, an effect of WRB knockout on CAML mRNA was not observed in our system suggesting that when WRB is missing the major effect is on turnover of the CAML protein.

The analysis of populations of isolated cardiomyocytes revealed residual WRB signals at both the mRNA (Fig. 1b) and the protein level (Fig. 1c). This is unlikely to be due to incomplete recombination^34^ and may well be explained by the presence of contaminating fibroblasts or endothelial cells in the primary cell isolation. As the stability of the WRB protein in isolated adult cardiomyocytes is currently unknown we cannot fully exclude the possibility that some cells retained some WRB protein at the tested time point after knockout induction. Importantly, we observed that the steady-state levels of not only the CAML but also the TRC40 protein were strongly reduced upon cardiomyocyte-specific knockout of WRB.

### The steady-state levels of some cardiomyocyte TA proteins are reduced

Next, we analyzed the steady-state levels of a subset of TA proteins by western blot and found that syntaxin 5 (Stx5), an essential SNARE involved in Golgi-to-ER trafficking, and emerin (EMD), a TA protein of the inner nuclear membrane^37^, were significantly less abundant compared to littermates not expressing the recombinase (Fig. 1e,f). In contrast, steady-state protein levels of Sec61β, an auxiliary translocon subunit, and syntaxin 6 (Stx6) were not significantly changed and those of syntaxin 8 (Stx8), an endosomal SNARE, not at all affected. Likewise, there was no effect on the steady-state protein levels of a polytopic control membrane protein, STT3B, a subunit of the protein glycosyltransferase. Quantitative analysis of the transcripts of the tested TA proteins (Supplementary Fig. 1) excluded that the variations observed at the protein level were caused by corresponding changes at the mRNA level. Tamoxifen-injected animals of the same age from a control mouse line carrying the MerCreMer recombinase gene and wild type WRB alleles did not show major alterations in the protein levels (Supplementary Fig. 2a–d), excluding side effects of tamoxifen injection, MerCreMer expression, or their combination as the cause of our observations.

### CAML and TA protein levels are reduced in WRB ko hepatocytes

**Figure 2:**
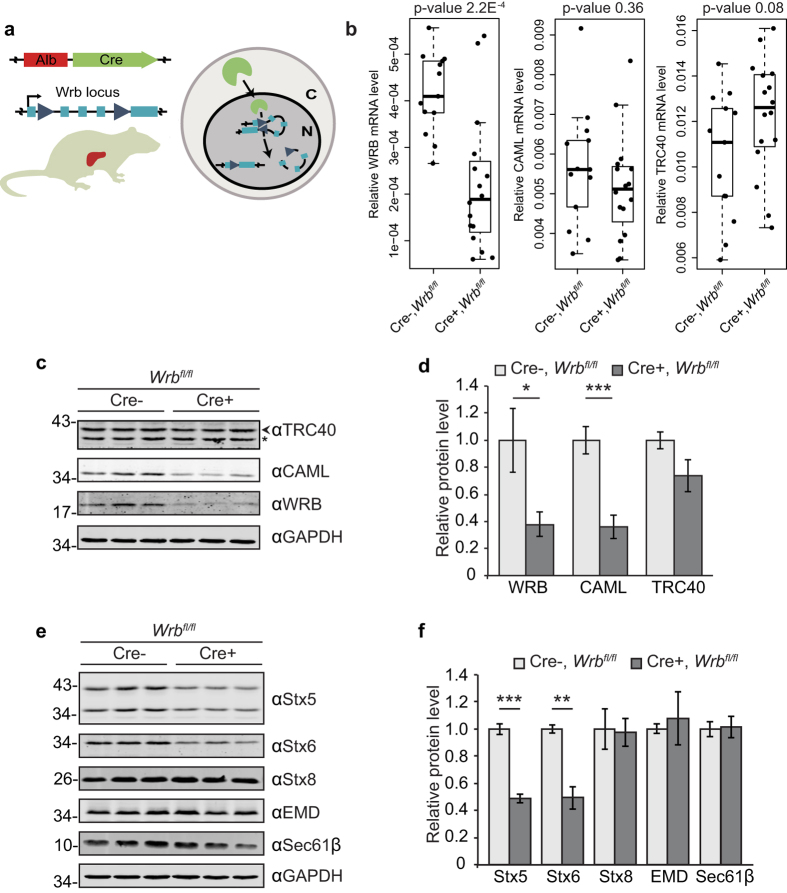
A hepatocyte-specific knockout of WRB leads to a reduction of the TRC40 pathway membrane receptor and substrate TA proteins. (**a**) Schematic of the hepatocyte specific knockout of WRB. Cre recombinase is expressed under the control of albumin promoter in a WRB^fl/fl^ mouse line, allowing recombination of the WRB locus (**b**) mRNA levels of WRB, CAML and TRC40 were analysed by qPCR. Boxplots depict the levels relative to GAPDH in six-week old Alb-Cre negative and positive littermates. Y-axes show 2^−ΔCP^ values. p-values are indicated. (**c**) Protein lysates from isolated hepatocytes were separated by SDS-PAGE and expression level of the main pathway components were evaluated by western blot. Blots of knockout animals in the 25^th^ percentile of the boxplot of WRB mRNA levels in **b**, and of control animals are shown. GAPDH was used as loading control. An asterisk marks a cross-reactive protein. (**d**) Quantification of the blots from (**c**). Bars represent average −/+s.e.m. (n = 4, *p-value < 0.05; ***p-value < 0.001). (**e**) Lysates from isolated hepatocytes were separated by SDS-PAGE and expression level of known substrates of the pathway was evaluated by western blot. Blots of knockout animals in the 25^th^ percentile of the boxplot of WRB mRNA levels in **b**, and of control animals are shown. GAPDH was used as loading control. (**f**) Quantifications of the blots from e. Bars represent average −/+s.e.m. (n = 4, **p-value < 0.01; ***p-value < 0.001).

To generate a liver-specific WRB knockout, we crossed the same WRB floxed line, with a line expressing Cre recombinase under the control of the constitutively active albumin promoter^38^ (Fig. 2a). In our qPCR analysis, isolated hepatocytes showed a heterogeneous response to recombination. Reduction of WRB mRNA ranged from 5% to 90% (Fig. 2b), potentially due to the regeneration capacity of adult hepatocytes^39^ and clonal expansion of cells without the recombined transgene. Independently of the reduction level, all Cre-positive animals showed severe liver damage that was macroscopically visible upon dissection of the animals, and was corroborated by histological sections revealing fibrous areas and by elevated plasma levels of liver enzymes ALT and AST (Supplementary Fig. 4a,b). These strong general effects on the integrity of the liver support previous observations of the essential functions of TRC40 pathway in development and tissue homeostasis^21^,^22^,^23^,^25^,^29^,^30^. In contrast to enzymatic activities indicating liver damage, the serological levels of cholesterol and triglycerides in Cre-positive animals did not change significantly in comparison to control littermates (Supplementary Fig. 4c), indicating sufficient residual liver function.

As in cardiomyocytes, no major differences were observed in the mRNA levels of CAML and TRC40 in hepatocytes (Fig. 2b). We observed that only the few animals that displayed a reduction in WRB mRNA of 70–90%, showed a significantly reduced protein level of both WRB and CAML, whereas TRC40 levels were not changed significantly (Fig. 2c,d). In addition, these animals confirmed the altered steady-state levels of a subset of TA proteins including Stx5 and Stx6 (Fig. 2e,f) that was not caused by changes at the mRNA level (Supplementary Fig. 1), in accordance with the results obtained in cardiomyocytes (Fig. 1e,f). Animals with a weak reduction of WRB mRNA showed insignificant variations of WRB, CAML and TA protein levels (Supplementary Fig. 4d,e), which may suggest a threshold dependence of the knockout effects.

### Sensitive TA proteins mislocalize in cardiomyocytes and hepatocytes

**Figure 3:**
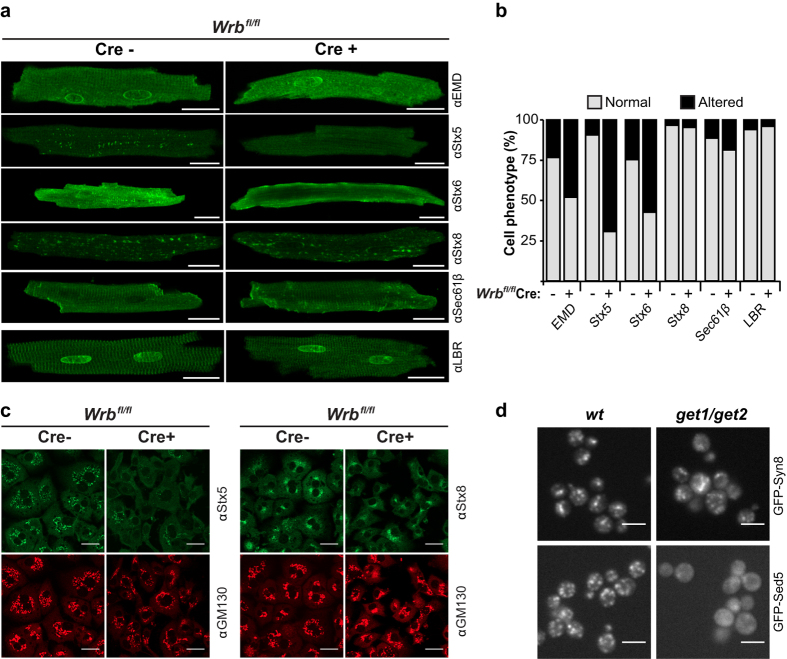
Depletion of the TRC40 pathway receptor results in mislocalization of a subset of TA-proteins *in vivo*. (**a**) Ventricular cardiomyocytes from eight-week old MerCreMer- (control) and MerCreMer+ (KO) Wrb^fl/fl^ littermates two weeks after tamoxifen induction were isolated and subcellular localization of selected TA proteins (EMD, Stx5, Stx6, Stx8, Sec61β) was analysed by indirect immunofluorescence. Images were acquired with a confocal microscope. The multi-spanning membrane protein LBR served as control. Scale bar: 20 μm. (**b**) Quantification of mislocalization phenotype. For each protein, 22 to 98 cells isolated from 4 to 8 animals were examined. The scoring was performed blindly by three investigators using an image shuffling pipeline and automated genotype/phenotype decoder. The following criteria were applied to assign an “altered” phenotype: for Stx5, Stx6 and Stx8 loss of staining at membraneous structures resembling Golgi or endosomes; for Sec61β loss of staining at cellular striations resembling the sarcoplasmic reticulum; for emerin and LBR loss of staining at the nuclear rim and sarcoplasmic reticulum striations. (**c**) Isolated hepatocytes from six-week old animals were immunostained for either Stx5 or Stx8 and images were acquired with a confocal microscope. GM130 was used as a Golgi marker. Scale bar: 20 μm. (**d**) Live-cell microscopy of wild type and *get1/get2* yeast cells expressing GFP-Sed5 or GFP-Syn8. Scale bar: 5 μm.

In order to investigate the subcellular localization of client TA proteins as a proxy for successful membrane targeting, we performed indirect immunostaining of isolated cardiomyocytes and hepatocytes. Not all TA proteins studied were severely affected by WRB knockout with respect to their localization. In cardiomyocytes we observed a strong reduction of Stx5 and Stx6 in vesicular structures resembling the Golgi apparatus (Fig. 3a,b) as confirmed by co-staining with p115 (Supplementary Fig. 3). In about 50% of the cells, emerin was less clearly localized to the striated cardiomyocyte ER and the nuclear envelope. No effect was observed for the well studied TRC40 substrate Sec61β^3^,^4^ and Stx8 (Fig. 3a,b). Evaluation of the localization patterns described above was conducted blindly by three independent investigators using a computational pipeline that shuffles and presents the images without revealing the genotype and an automated decoder that provides the scoring results. Generally, the effects on subcellular localization were correlated with the steady-state protein levels of the tested TA proteins (Fig. 1e,f). TA protein localization was unaltered in Cre-negative or -positive littermate cardiomyocytes from non-floxed animals injected with tamoxifen and analyzed at the same time point after injection (Supplementary Fig. 2e). In knockout hepatocytes, Stx5 was also an example of a very strongly affected substrate as its Golgi localization was almost completely lost. For Stx8, a TA protein representing the other end of the spectrum based on the protein steady-state levels, we did not observe comparable alterations in targeting to membrane structures although the endomembrane system of Cre-positive hepatocytes appeared slightly perturbed in its overall morphology (Fig. 3c). We conclude that the effect of knocking out the TRC40 receptor leads to a spectrum of biogenesis defects *in vivo* depending on the TA protein client investigated. For some TA proteins such as the well-established TRC40 client Sec61β, the effects were mild whereas some but not all of the syntaxins investigated were severely affected. Indeed, our results highlight Stx5 and Stx8 as examples of the respective extremes of TRC40 receptor dependence *in vivo* and implicate them as useful model proteins for the dissection of the protein features underlying differential TRC40-pathway dependence of TA protein clients.

### A systematic yeast screen reveals only 2 strictly GET-dependent clients

In our analysis of endogenous TA proteins in differentiated mammalian cells, the TA proteins that were identified as strictly dependent on the TRC40 pathway were not dedicated to specific cellular functions (such as vesicle fusion) and did not belong to a certain protein family (such as the SNAREs). On the contrary, strong sensitivity of selected clients (*e.g.* Stx5 or emerin) may depend on unique features specific to each TA protein. In order to extend our analysis to a greater number of TA proteins we turned to yeast, and performed a systematic screen of yeast TA proteins to determine which substrates are affected in cells lacking the GET pathway^10^.

From a recently generated SWAp-Tag yeast library^40^, we assembled a subset of yeast strains expressing N-terminally GFP-tagged TA proteins under the control of an intermediate strength, constitutive promoter (*NOP1*pr). The selection was conducted according to a list of predicted human TA proteins^17^, from which we extracted the 46 with known yeast homologues. We generated deletions of GET pathway genes in all 46 strains, either the double deletion *get1/get2* (no GET receptor), single *get3* deletion (no cytosolic targeting ATPase), or triple *get1/get2/get3* deletion (no GET pathway) and analyzed them by fluorescence microscopy. This analysis revealed that only two out of the 46 TA proteins were severely affected by deletion of the GET pathway (Fig. 3d and Supplementary Fig. 5a–c). One of the two mislocalized TA proteins was the yeast homolog of Stx5, the well studied SNARE protein Sed5^10^,^36^,^41^ and the other Lam5, a member of a recently characterized family of lipid transfer proteins^42^. Absolute expression levels, as quantified by flow cytometry measuring the fluorescence intensity of the tested GFP fusion proteins, revealed minor changes for Sed5, Sbh1 and Lam5 in GET mutants and absence of variation for Syn8 (Supplementary Fig. 5b). Western blot assessment of the steady-state levels of endogenous Sed5 implied that the mistargeted protein remained stable (Supplementary Fig. 5d,e), possibly due to the action of various chaperones^43^. Importantly, Syn8, the yeast homolog of Stx8, was unaffected (Fig. 3d and Supplementary Fig. 5a,b) as were the 43 other TA proteins screened, including TA proteins that were previously shown to use the GET pathway *in vitro* such as Sec22^10^. Other yeast TA proteins are known to be mislocalized in cells lacking the GET receptor upon over-expression from a strong inducible promoter, such as Sbh1 (homolog of Sec61β) and Ysy6 (homolog of mammalian Ramp4, also demonstrated to depend on TRC40 for targeting *in vitro*^4^). However, in our strains the tested TA proteins were expressed at levels closer to the endogenous situation and this may explain why they did not show significant variations in the screen. Importantly, the systematic yeast screen confirms a graded dependence of clients on the GET pathway with Stx5 (Sed5) and Stx8 (Syn8) representing the ends of the spectrum.

### Stx5 is not stabilized by proteasomal inhibition

**Figure 4:**
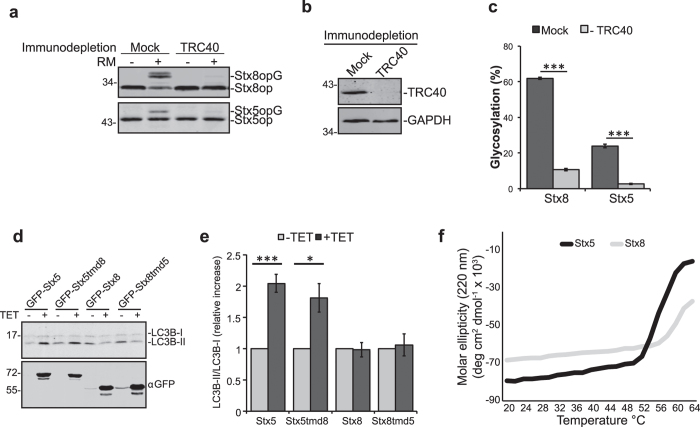
The cytoplasmic domain of Syntaxin 5 is aggregation-prone and can induce autophagy. (**a**) Stx5op and Stx8op were translated *in vitro* in a rabbit reticulocyte lysate or in a lysate immunodepleted of TRC40. (**b**) Integration into rough microsomal membranes (RM) was detected by glycosylation of the C-terminal opsin tag (opG) resulting in a slower-migrating protein detected by an opsin-specific antibody. (**c**) Quantification of glycosylation, percentage of total. Bars represent average +/−s.e.m. (n = 4, ***p-value < 0.001). (**d**) Stably transfected HEK Flp-In T-REx cells were cultured in absence or presence of tetracycline for 16 hours to induce expression of Stx5/Stx8 constructs. Cellular lysates were separated by SDS-PAGE and induction was verified by anti-GFP western blot. An increased ratio between the lipidated (LC3B-II) compared to non-lipidated (LC3B-I) LC3B was evaluated by blot and quantified in **e** Bars represent average +/−s.e.m. (n = 4, *p-value < 0.05; ***p-value < 0.001). (**f**) Far-UV circular dichroism measured at 220 nm and increasing temperatures for MBP-Stx5cyt (black trace) and MBP-Stx8cyt (gray trace).

It has been proposed that strongly hydrophobic transmembrane domains strictly require TRC40-mediate targeting whereas more hydrophilic ones can be substrates of alternative pathways^44^. Our results show that Stx5 is more dependent on the TRC40 pathway despite its transmembrane domain being less hydrophobic than that of Stx8 (47.6 and 61.3 respectively according to the Kyte-Doolittle scale^45^). We tested Stx5 and Stx8 in an *in-vitro* transcription/translation reaction coupled with insertion into canine pancreatic rough microsomes (RM). When the reaction was performed using rabbit reticulocyte lysate depleted of TRC40, membrane integration of both Stx5 and Stx8 was strongly inhibited (Fig. 4a–c). This result clearly shows that both proteins can be targeted by TRC40 *in vitro* and reveals that additional molecular mechanisms may govern TA protein biogenesis *in vivo*.

BAG6 is a component of the TA protein pre-targeting complex but is also known to be involved in the degradation of mislocalized proteins via the ubiquitin-proteasome system^6^,^7^. Together with our finding that Stx5 protein levels were lower in mammalian WRB knockout cells (Figs 1e,f and 2e,f) while those of Stx8 were nearly unchanged, this dual function of the BAG6-containing pre-targeting complex led us to test in HeLa cells whether Stx5 was targeted for proteasomal degradation in the absence of the TRC40 receptor. Cells were transfected with siRNA to specifically knock down WRB or TRC40 and were treated with the proteasomal inhibitor oprozomib (OPZ). No significant variations in steady-state levels were observed for Stx5 or Stx8 (Supplementary Fig. 6a,b) therefore suggesting that mistargeted Stx5 is not degraded by the proteasome.

### The cytoplasmic domain of Stx5 is prone to aggregation and induces autophagy

Another cellular process to degrade proteins or even entire organelles is autophagy^46^. We generated HEK293 cells allowing tetracycline-inducible overexpression of Stx5, Stx8, or chimeric proteins with swapped transmembrane domains and monitored autophagy induction via the relative levels of the lipidated form of LC3B, a known autophagosomal marker^47^. Overexpression of Stx5 or a variant of Stx5 with the transmembrane domain (TMD) of Stx8 (Stx5tmd8), but not full-length Stx8 or the inverse chimera, significantly increased the ratio of lipidated versus non-lipidated LC3B, indicating increased levels of autophagosomal membranes (Fig. 4d,e). This result strongly suggests that physicochemical properties of the cytoplasmic domain of Stx5 are responsible for the observed autophagy induction.

As autophagy is responsible for the clearance of protein aggregates, we wondered if the cytoplasmic domain of Stx5 has a higher tendency to misfold or aggregate compared to that of Stx8. We purified the cytoplasmic domains of Stx5 (333 amino acids) and Stx8 (216 amino acids) as MBP-tagged recombinant proteins and performed circular dichroism (CD) spectroscopy to monitor changes in secondary structures upon heating. We found that the cytoplasmic domain of Stx5 had a higher thermal instability and unfolding propensity relative to the same domain in Stx8 (Fig. 4f). An *in-vitro* aggregation assay confirmed these findings by showing that MBP-Stx5cyt incubated at 42 °C and 55 °C appeared in the insoluble pellet after centrifugation whereas MBP-Stx8cyt remained in the soluble fraction (Supplementary Fig. 6c).

We also detected a strong accumulation of TRC40 and a mild but significant stabilization of Stx5 in cells treated with chloroquine (CQ), a drug that inhibits lysosomal proteases by preventing acidification of lysosomes^48^. In contrast, variation in Stx8 steady-state levels was not significant (Supplementary Fig. 6d–g). These results indicate that Stx5 is not only a substrate of lysosome-mediated degradation but can also induce autophagy through distinct properties of its cytoplasmic domain.

### FAM134B is involved in Stx5 degradation

In order to confirm an active role for autophagy in the clearance of Stx5, we used siRNA to selectively down regulate in HeLa cells two autophagy cargo receptors: FAM134B and p62/SQSTM1. FAM134B was recently identified as a member of the reticulon protein family involved in turnover and degradation of the ER and membrane-associated proteins by autophagy^49^. p62/SQSTM1 is a known cargo receptor that mediates autophagosomal degradation of ubiquitinated and non-ubiquitinated cytosolic aggregates^50^.

**Figure 5:**
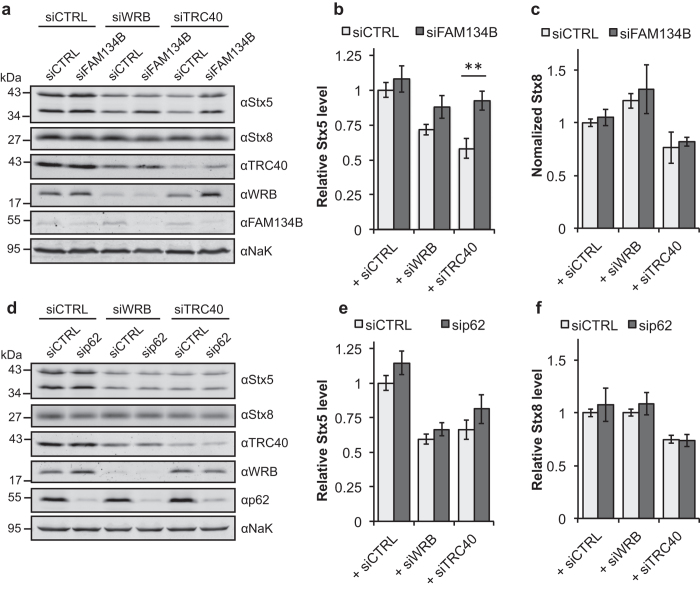
Stx5 is degraded by a FAM134B-dependent autophagy mechanism. (**a**) Control-silenced HeLa cells or knockdown cells for WRB or TRC40 were co-transfected with a control siRNA or with a FAM134B-specific siRNA. Cellular lysates were analyzed by Western blot for the indicated proteins. Graphs in (**b**,**c**) show quantification of the expression levels of Stx5 and Stx8 respectively. Bars represent average +/−s.e.m. (n = 6, **p-value < 0.01). (**d**) Control-silenced HeLa cells or knockdown cells for WRB or TRC40 were co-transfected with a control siRNA or with a p62-specific siRNA. Cellular lysates were analyzed by Western blot for the indicated proteins. Graphs in **e,f** show quantification of the steady-state levels of Stx5 and Stx8 respectively. Bars represent average +/−s.e.m. (n = 4, variations not significant).

We observed a significant stabilization of Stx5 in cells silenced for TRC40 in combination with FAM134B (Fig. 5a,b). Stx8 was not affected by down-regulation of FAM134B (Fig. 5a–c). p62/SQSTM1 silencing stabilized neither Stx5 nor Stx8 (Fig. 5d–f). These results suggest the existence of a mechanism of Stx5 degradation after its membrane integration in a FAM134 dependent manner.

## Discussion

The GET/TRC40 pathway was discovered by a combination of experimental strategies using *in-vitro* biochemical dissection in mammalian lysates, structural biology, and the model organism *S. cerevisiae*^3^,^4^,^10^,^18^. These studies identified the key components of the pathway in the cytosol and at the ER membrane. They were also instrumental in demonstrating the interaction of Get3/TRC40 with client TA proteins, with the pre-targeting complex and its ER receptor. These approaches have culminated in a detailed mechanistic model of the targeting process. However, only a small selection of client TA proteins was studied based on practical aspects such as efficient translation in extracts, antibody availability, or ease of recombinant expression and purification. In consequence, the actual client spectrum that depends on the pathway *in vivo* is unknown and so are the properties of clients that make them GET/TRC40 pathway dependent in their targeting.

In higher eukaryotes, TRC40 and the subunits of its receptor WRB and CAML have been investigated based on screens that identified them as being involved in sensory processes^25^,^26^,^31^,^32^. These physiological studies dissect the *in-vivo* function of the GET/TRC40 pathway proteins from the behavioral phenotype to cellular morphology and detect an impairment of the synaptic endomembrane system that is required for sensory transduction. Interestingly, it was demonstrated that the most critical substrate of WRB-dependent targeting in hair cells of the inner ear is otoferlin. In zebrafish and in mouse, hair-cell specific knockout of WRB lead to hearing deficits that phenocopy loss of otoferlin and can be rescued by overexpression of this TA protein in zebrafish^26^.

We investigated tissue-specific WRB knockout mice to analyze the steady-state levels and subcellular localization of a set of TA proteins including well-established model TA proteins such as Sec61β and different SNAREs in two biochemically amenable differentiated cell types, cardiomyocytes and hepatocytes. We find a graded response of different TA protein clients to loss of the TRC40 receptor with some being much more destabilized and mislocalized than others. *In-vitro* studies^44^,^51^ have suggested alternative targeting mechanisms involving cytosolic chaperones such as Hsc70/Hsp40 or SRP and additional SRP-independent targeting pathways for TA proteins may exist^19^,^52^. Nevertheless, the properties of a substrate that determine its strict dependence on a specific pathway are still unknown.

Together with a recent study showing that Stx5 and Stx6 are critically affected TA proteins in pancreatic beta cells lacking TRC40^23^, our results identify Stx5 as one of the substrates that strictly depends on the TRC40 pathway for its biogenesis. This dependence is conserved in the yeast system and therefore reflects a property of the client protein. We exploited the fact that another SNARE, Stx8, was not affected by loss of WRB in a chimeric approach and found that the cytoplasmic domain of Stx5 stimulated autophagy in cells overexpressing constructs that included this part of the TA protein irrespective of the transmembrane domain on which it was presented. It has been hypothesized that very hydrophobic transmembrane domains require TRC40 whereas more hydrophilic ones can be substrates of alternative pathways^44^ or capable of spontaneous insertion^53^. Our findings suggest that although properties of the transmembrane domain of TA proteins might be relevant to drive targeting and to ensure the fidelity of membrane integration, the cytoplasmic domain may be even more important for the fate of the protein during biogenesis *in vivo*.

Accumulation of protein aggregates is a metabolic vulnerability for cells that have to ensure rapid clearance of these aggregates. When membrane targeting via the TRC40 pathway is impaired, due to loss of WRB, the flow of aggregated or misfolded Stx5 and hence its turnover by autophagy increases. Our results indicate a role of FAM134B in Stx5 clearance. This protein was recently shown to have an essential function in the turnover of ER by autophagy^49^. Mice lacking FAM134B display an increased ER volume and structure but also a fragmented and elongated Golgi apparatus^49^.

As silencing of FAM134B stabilizes Stx5 on protein level (Fig. 5a,b) it is conceivable that a certain proportion of Stx5 is inserted into membrane by alternative targeting mechanisms even in absence of a functional TRC40 pathway. Nevertheless, the observation that Stx5 is extremely reduced in absence of WRB or TRC40 raises the possibility that Stx5 targeted to membrane via alternative pathway may be not fully functional. The presence of a non-functional SNARE protein may lead to its active removal and degradation by autophagy. This could be due to many reasons. In the absence of the TRC40 pathway, the targeting fidelity of alternative targeting mechanisms may not support targeting to the right ER domain. It is also conceivable that the TRC40 pathway might mediate the biogenesis of a factor that is essential for the stability and function of Stx5. Another possibility is that Stx5 is so exquisitely sensitive to loss of the TRC40/GET pathway because it requires the chaperone holdase activity of TRC40/Get3^20^,^43^ in addition to the TA protein targeting function.

In general, our results indicate that the biogenesis of the vast majority of TA proteins is not acutely affected by deletion of the TRC40/GET pathway *in vivo*. Indeed, decreasing the number of available receptor sites *in vitro*^18^ or overexpressing a dominant negative mutant of TRC40 in transfected mammalian cells^54^ slows down membrane integration and increases the amount of stalled TA proteins in the cytosol. Cells may then cope with accumulating TA proteins by promoting their membrane integration via alternative pathways or by ensuring the degradation of clients prone to aggregate or to cause proteotoxic stress (i.e. Stx5). Therefore, it may not be appropriate to reduce the model of TA protein biogenesis to a mere sequence of recognition-targeting-integration events. Instead, it may be necessary to consider the process in the context of the cellular proteostatic network that includes additional mechanisms of quality control and degradation. In this view, the extent to which a TA protein is impaired in its biogenesis by loss of the TRC40/GET pathway will depend on the properties of the whole protein, cytoplasmic domain and transmembrane segment, as well as its abundance in a given cell type. Further tissue specificity will arise from the nature of the pertinent cellular proteostasis network (components and their abundance). The phenotypes observed upon TRC40 pathway knockout in the diverse cell types of multicellular organisms will reflect the most vulnerable and important TA proteins in that cell – as illustrated by the consequences of impairing Stx5/6 biogenesis in pancreatic beta cells for insulin secretion^23^ or by the defects in otoferlin targeting observed in the WRB inner hair cell knockout^26^. These results expand and refine our knowledge on the TRC40 pathway in eukaryotes and provide a first view on its integration into tissue physiology.

## Methods

### Cell lines

HeLa P4 cells were grown in DMEM (Gibco) supplemented with 10% (v/v) FBS (Gibco). Plasmid and siRNA transfections were performed with BBS-calcium phosphate^55^. For siRNA-mediated knockdowns, cells were transfected twice at 48 hours interval, using RNA oligos specific for WRB (sense AAAUCCAACAGGUAAUUCCAACACC, antisense GGUGUUGGAAUUACCUGUUGGAUUU, Invitrogen), TRC40 (s1675, Ambion), FAM134B (sense AGGUAUCCUGGACUGAUAAUG, antisense CAUUAUCAGUCCAGGAUACCU, Sigma Aldrich), p62/SQSTM1 (sense GCAUUGAAGUUGAUAUCGAU, antisense AUCGAUAUCAACUUCAAUGC, Sigma Aldrich), and non targeting control siRNA (AM4635, Ambion).

Hek Flp-In T-REx were cotransfected with combinations of the recombinase containing vector pOG44 and the pCDNA5-FRT-TO-N-GFP constructs described in the “Plasmids” section. Recombined clones were selected for resistance to Hygromycin B (150 μg/ml). Expression of Stx5, Stx8 or chimeric proteins was induced by adding 1 μg/ml tetracycline to cell medium for 16 hours.

### Mice

All the procedures involving animals were reviewed and approved by the Institutional Animal Care and Use Committee of the University Medical Center Göttingen, in compliance with the humane care and use of laboratory animals.

WRB^fl/fl^ line was previously described^26^. Myh6-MerCreMer (B6.FVB(129)-Tg(Myh6-cre/Esr1*)1JmK/J) and Albumin-Cre mice (B6.Cg-Tg(Alb-cre)21Mgn/J) were purchased from the Jackson Laboratory.

MerCreMer dependent recombination was induced in six-week old animals by injection of 40 mg/kg of tamoxifen (diluted in EtOH-soybean oil) as previously described^34^.

### Genotyping

Mice tails were harvested before three weeks of age, and lysed for 14–16 hours at 56 °C in DirectPCR-Tail Lysis reagent (Peqlab), plus proteinaseK (20 μg/mL), followed by inactivation at 85 °C, 45 min. Supernatant was used as PCR template, with MangoTag (Bioline) and primers for wt and transgene as shown in “Primers” Table. Products were then analysed with standard agarose gel electrophoresis.

### Primary cells isolation and culture

At 8 weeks of age and two weeks after tamoxifen induction of the MerCreMer recombinase, ventricular cardiac myocytes were isolated by retrograde perfusion with a modified Langendorff^35^ setup with minor changes. Briefly, hearts were perfused at a rate of 4 ml/min with Ca^2+^ free oxygenated perfusion buffer for 4 min, 37 °C, followed by perfusion with collagenase type II (600 U/ml in perfusion buffer supplemented with 12.5 μM CaCl_2_, Worthington Biochemical Corporation) for 8 min at 37 °C. Digested ventricles were minced in collagenase-containing buffer and the cardiomyocytes were released by gentle pipetting. Digestion was stopped with 10% bovine calf serum in perfusion buffer, and the cells were washed twice in the same buffer. The quality of the preparations was verified in a Neubauer chamber, and isolations with less than 60% rod shape cells were discarded. For indirect immunofluorescence microscopy, isolated cardiomyocytes were plated on laminin (BD Biosciences)-coated glass coverslips.

Primary hepatocytes were isolated from six-week old animals by perfusing livers with collagenase and subjecting the isolated cells to a Percoll gradient. Briefly, the liver was perfused via the *Vena cava caudalis*, using the *Vena portae* as outflow with 125 mL Krebs-Ringer/EGTA, followed by perfusion with Krebs-Ringer substituted with Collagenase/HEPES/CaCl_2_ for 4 min. After perfusion, the liver was excised, rinsed in Williams E (Gibco) and pulled apart with forceps to wash out the cells, followed by a filtering step (70 μm pore size). The cells were then subjected to a Percoll gradient (GE Healthcare). Hepatocytes were washed once and resuspended in Williams E (1 g of cells in 50 mL). The amount of cells was quantified using a counting chamber. For indirect immunofluorescence, cells were plated on collagen-coated glass coverslips.

### Blood withdrawal

Animals were anesthetized with isoflurane followed by injection of approx. 400 μL Nembutal (10 mg/mL). Blood was taken from the *Vena cava caudalis* with a small syringe after opening the body cavity. For obtaining serum for the analysis, the syringe was rinsed with lithium-heparine (Sigma-Aldrich) before drawing blood. Samples were then centrifuged at 500 g for 30 min to collect the serum. Blood analysis was carried out by the Department of Clinical Chemistry of the University Medical School Göttingen.

### Histology

Livers were embedded in Paraffin and 3 μm sections were obtained at a microtome. Sections were stained with hematoxylin-eosin or Goldner staining kit (Engelbrecht).

### qPCR

mRNA was isolated from samples using a Roche High Pure RNA isolation kit, following manufacturer’s instructions. Equal amounts of total RNA were subjected to cDNA synthesis (SuperScript III First-Strand Synthesis System). qPCRs were performed using a Roche SYBR green Master Mix in a Roche Lightcycler480. mRNA levels were normalized to GAPDH mRNA.

### Protein extraction and Western Blot

For mammalian samples, equal amounts of cells were lysed in solubilization buffer (50 mM Tris-HCl pH7.4, 10 mM NaCl, 5 mM EDTA, 2.5 mM EGTA, 1.5% Triton X-100, 0.75% Na-deoxycholate, 0.1% SDS) supplemented with protease inhibitors. Proteins were precipitated by TCA before SDS-PAGE separation and immunoblot analysis.

For yeast protein extraction, cells were recovered by low speed centrifugation and resuspended in 100 mM NaOH for 10 minutes. Cells were pelleted and lysed in SDS loading buffer.

Primary and secondary antibodies were diluted (as described in the “Antibodies” Table) in blocking buffer (5% milk in PBS, 0.1% Tween). Blots were imaged using an Odyssey Sa Infrared imaging system with IRDye LiCOR secondary antibodies. Quantification was performed using the ImageStudio Software (LI-COR).

### Indirect immunofluorescence microscopy

For immunofluorescence, cells were fixed as described in the “Antibodies” Table. Samples were blocked with 10% FCS in PBS for 30 min and incubated with primary antibodies diluted in blocking buffer at 4 °C overnight. Incubation with Alexa Fluor secondary antibodies (Thermo Fisher Scientific) was performed for 1 hour at room temperature, and the samples were mounted with Mowiol-DAPI.

Images were taken using a LSM 510-META confocal laser scanning microscope (Zeiss) with a 63x Plan-Neofluar 1.3 NA water-corrected objective and appropriate filter settings. Images were processed using ImageJ software (https://imagej.nih.gov/ij/).

### Blind annotation KNIME workflow

Using a KNIME workflow, images were copied and given a random name after which they were manually annotated. The results of the annotation were decoded using the same workflow. The workflow was written in KNIME 3.2.1 (https://www.knime.org) and requires the KNIME File Handling Nodes (3.2.2.v201609201941) plugin.

### Yeast strains

Yeast strains were selected from the SWAp-Tag library^40^. SGA crosses were carried out as described previously^56^ by crossing the strains with a query strain (MATα *his3*∆1 *leu*2∆0 *met15*∆0 *ura3*∆0 *can1*∆::*STE2*pr-sp*HIS5* *lyp1*∆::*STE3*pr-*LEU2*) carrying combinations of *get1∆::*KAN, *get2∆::*NAT, and *get3∆::*BLE to yield *get1/get2, get3,* and *get1/get2/get3* strains, as indicated. The query strains with and without *GET1/GET2/GET3* replacements were also used for Western blots, as indicated.

### Live-cell imaging

Logarithmic phase cultures of indicated yeast strains grown in synthetic dropout media were diluted and transferred into BioConext (United Chemical Technologies) and Concanavalin A (Sigma-Aldrich) coated 384-well glass-bottom plates (Matriplate, Brooks Life Science Systems). Microtiter plates were automatically imaged at 30 °C on an Imaging Machine 03-dual (Acquifer) widefield high content screening microscope, equipped with a white LED array for bright field imaging, an LED fluorescence excitation light source, an sCMOS (2048 × 2048 pixel) camera, a temperature-controlled incubation chamber, and a stationary plate holder in combination with movable optics. Images were acquired with 470 nm filter cubes (Ex 469/35, Em 525/39, dichroic 497) using 3 z-slices (dz = 1 μm) and a 40x CFI Super Plan Fluor ELWD N.A. 0.60 (Nikon), using 500 ms integration time. The focal plane was detected in the bright field channel using a yeast autofocus algorithm.

### Flow cytometry

Yeast strains were cultured in synthetic complete media at 30 °C overnight, diluted to OD_600_ 0.2, cultured for 4 hours at 30 °C, washed and resuspended in PBS. Fluorescence intensity was measured with a BD FACSCanto flow cytometer (BD biosciences). Data were analyzed with custom R scripts using the Bioconductor package FlowCore^57^. GFP fluorescence intensities were represented as Kernel density plots using the ggplot2 graphing package^58^.

### *In vitro* transcription/translation

Stx5op and Stx8op were synthesized using a TnT Quick Coupled Transcription/Translation System (Promega) according to manufacturer instructions. Briefly, 200 ng of plasmid containing the coding sequence of the protein of interest were incubated with 10 μl of reaction mixture. After 45 minutes, rough microsomes were added and the reaction was incubated for 45 more minutes. Immunodepletion of TRC40 was performed as previously described^59^.

### Protein purification

pQE80-MBPtevStx5cyt and pQE80-MBPtevStx8cyt were transformed into E. coli BL21AI. Induction of recombinant proteins was performed at 30 °C by addition of 0.5 mM IPTG for 2 hours. Cells were lysed in PBS with an Avestin-Emulsiflex and lysates cleared by centrifugation at 100,000 g for 45 minutes. The lysates were incubated with an amylose resin (NEB) for one hour at 4 °C. The resin was washed three times with ten volumes of PBS and finally the proteins eluted in PBS supplemented with 20 mM maltose.

### Aggregation assay and circular dichroism spectrocopy

10 μM of MBPtevStx5cyt or MBPtevStx8cyt were incubated at different temperatures (as shown in Supplementary Fig. 6c) for one hour. Insoluble material was separated by centrifugation at 25,000 g for 20 minutes. Fractions of soluble and insoluble proteins were analysed by SDS-PAGE and stained by coomassie blue.

Changes in the secondary structure of the cytosolic domains of Stx5 and Stx8 were determined by recording far-UV CD spectra at 2 °C temperature intervals (from 20 °C to 62 °C) using a Chirascan qCD (Applied Photophysics). For these measurements, proteins were dialyzed against CD buffer (30 mM Na-phosphate pH 7, 300 mM NaF, 0.1 mM TCEP) and diluted to 10 μM.

## Additional Information

**How to cite this article**: Rivera-Monroy, J. *et al*. Mice lacking WRB reveal differential biogenesis requirements of tail-anchored proteins *in vivo. Sci. Rep.* **6**, 39464; doi: 10.1038/srep39464 (2016).

**Publisher's note:** Springer Nature remains neutral with regard to jurisdictional claims in published maps and institutional affiliations.

## Supplementary Material

Supplementary Figures

Supplementary Information
