# Supplementary material for: Mice lacking WRB reveal differential biogenesis requirements of tail-anchored proteins in vivo
Source: Sci Rep. 2016 Dec 21;6:39464. doi: 10.1038/srep39464 (PMC5175141; doi:10.1038/srep39464)

Supplementary Information

Mice lacking WRB reveal differential biogenesis requirements of tail-anchored proteins *in vivo*

Jhon Rivera-Monroy[+], Lena Musiol[+], Kirsten Unthan-Fechner, Ákos Farkas, Anne Clancy, Javier Coy-Vergara, Uri Weill, Sarah Gockel, Shuh-Yow Lin, David P Corey, Tobias Kohl, Philipp Ströbel, Maya Schuldiner, Blanche Schwappach[*] and Fabio Vilardi[*]

[+] These authors contributed equally to this work.

[*] Correspondence should be addressed to

blanche.schwappach@med.uni-goettingen.de and

fabio.vilardi@med.uni-goettingen.de

Materials

Plasmids

For pGem3Z-Stx5op, the coding sequence of Stx5 was amplified from heart cDNA using primers #1 and #2. The KpnI/HindIII fragment was then cloned into pGEM3Z (Promega). pGem3Z-Stx8op was obtained similarly using primers #3 and #4

For pQE80-MBP-Stx5cyt, a KpnI/XhoI fragment was obtained using pGem3Z-Stx5op as template and primers #1 and #5. The fragment was cloned into pQE80-MBPtev[1]. pQE80-MBP-Stx8cyt was obtained similarly using pGem3Z-Stx8op as template and primers #3 and #6.

pCDNA5-FRT-TO-GFPStx5 was obtained by amplification of pGem3Z-Stx5op template with primers #1 and #11. The KpnI/XhoI digested fragment was cloned into pCDNA5-FRT-TO-N-GFP (Invitrogen). pCDNA5-FRT-TO-GFPStx8 was obtained similarly using pGem3Z-Stx8op as template and primers #2 and #11.

The chimeric Stx5-Stx8 constructs were obtained by fusion PCR.

For pCDNA5-FRT-TO-GFPStx5tmd8 the coding sequence of the cytosolic domain of Stx5 was amplified from pGem3Z-Stx5op using primers #1 and #7 whereas the region encoding the transmembrane segment of Stx8 was amplified from pGem3Z-Stx8op using primers #8 and #11. The PCR products were mixed in equimolar ratio and used as template for the fusion PCR using primers #1 and #11. A KpnI/XhoI fragment obtained from this final PCR was cloned into pCDNA5-FRT-TO-N-GFP.

For pCDNA5-FRT-TO-GFPStx8tmd5 the coding sequence of the cytosolic domain of Stx8 was amplified from pGem3Z-Stx8op using primers #3 and #9 whereas the region encoding the transmembrane segment of Stx8 was amplified from pGem3Z-Stx5op using primers #10 and #11. The PCR products were mixed in equimolar ratio and used as template for the fusion PCR using primers #1 and #11. A KpnI/XhoI fragment obtained from this final PCR was cloned into pCDNA5-FRT-TO-N-GFP.

Supplementary Reference

1. Favaloro, V., Vilardi, F., Schlecht, R., Mayer, M. P. & Dobberstein, B. Asna1/TRC40-mediated membrane insertion of tail-anchored proteins. *J. Cell. Sci.* **123,** 1522–1530 (2010).

Supplementary Table 1: Primers

#	Name	Sequence (5'-3')	Application
1	KpnI-Stx5-F	ATACTAGGTACCATGATCCCGCGGAAACGC	cloning
2	Stx5op-HindIII-R	TGATATAAGCTTTCAGCCCGTCTTGTTGGAGAAAGGCACGTAGAAGTTT GGGCCGGCAAGGAAGACCAC	cloning
3	KpnI-Stx8-F	TATACTGGTACCATGGCCCCAGACCCCTGG	cloning
4	Stx8op-HindIII-R	TGATATAAGCTTTCAGCCCGTCTTGTTGGAGAAAGGCACGTAGAAGTTT GGGCCGTTGGTTGGCCACACTGC	cloning
5	Stx5-Nterm-XhoI-R	ATACTACTCGAGTCACCGATTGGAGGTAAC	cloning
6	Stx8-Nterm-XhoI-R	ATACTACTCGAGTCACCCACAGGAAGCTGA	cloning
7	Stx5-Stx8-R	GCAATAAGATCACCATTATCATCCGATTGGAGGTAACTGACTGG	cloning
8	Stx5-Stx8-F	CCAGTCAGTTACCTCCAATCGGATGATAATGGTGATCTTATTGC	cloning
9	Stx8-Stx5-R	GGAAGATTTTGACCATGAGCCACCCACAGGAAGCTGACTTTCTG	cloning
10	Stx8-Stx5-F	CAGAAAGTCAGCTTCCTGTGGGTGGCTCATGGTCAAAATCTTCC	cloning
11	opsinSTOP-XhoI-R	TATACACTCGAGTCAGCCCGTCTTGTTGGAGAAAGG	cloning
12	mWRB-Ex1-F	AGCTTCGTGTTCGGGTGTAAC	qPCR
13	mWRB-Ex2-R	TTGACGGTGGACAGCTCCTG	qPCR
14	mWRB-Ex3/4-F	CTACATACTACAAGCAGCGCTGATG	qPCR
15	mWRB-Ex5-R	ATCCAACAGGTGATTCCAATTCCAC	qPCR
16	mTRC40-F	GTCCTTGTACGAGACGGAGC	qPCR
17	mTRC40-R	TTGCAGGGTTTCTCAGGGTC	qPCR
18	mCAML-F	CTCACCACGGCCTAGAACAG	qPCR
19	mCAML-R	AACTCTTCCGCGGTACTTCC	qPCR
20	mGAPDH-F	CCAATGTGTCCGTCGTGGATCT	qPCR
21	mGAPDH-R	GTTGAAGTCGCAGGAGACAACC	qPCR
22	mStx5-F	GCGGAAACGCTACGGATCTA	qPCR
23	mStx5-R	GAGTGCTGTCACTGCTGCTA	qPCR
24	mStx6-F	TCTCACGAGTTGGAGAGCAC	qPCR
25	mStx6-R	AGAGGATGGCTATGGCACAC	qPCR
26	mStx8-F	GGAGGGGGATCGAAGACAGA	qPCR
27	mStx8-R	GCTTCTTCGCTCATCAGGCT	qPCR
28	mEMD-F	GACCACCAAGACATACGGGG	qPCR
29	mEMD-R	AGTGTGCGATGCTCTGGTAG	qPCR
30	mSec61β-F	ATGTGGCGATTCTACACGGAA	qPCR
31	mSec61β-R	GCAGCATAAATACAGCAGCGA	qPCR
32	mSTT3B-F	CTCCAGTCCAAGTGTGGTCC	qPCR
33	mSTT3B-R	TGATAGCCGTAGTCCCACCA	qPCR
34	wt-Forward	TTGGTGAGGCTTGTCTCTGGCGATTT	Genotyping
35	wt-Reverse	GTTGATCTTCCTTTCCAGCCTGGCGTAT	Genotyping
36	dneo-Forward	AGTGTACACGCGTACTAGTCTAGCGAA	Genotyping
37	dneo-Reverse	ACTCAAAGGCAACAGCCAAGATAAGCA	Genotyping
38	oIMR3798	AGGTGGACCTGATCATGGAG	Genotyping
39	oIMR7338	CTAGGCCACAGAATTGAAAGATCT	Genotyping
40	oIMR7339	GTAGGTGGAAATTCTAGCATCATCC	Genotyping
41	oIMR8346	ATACCGGAGATCATGCAAGC	Genotyping
42	cre-only-F	ACTGACCGTACACCAAAATTTGC	Genotyping
43	cre-only-R	CCGCATAACCAGTGAAACAGC	Genotyping
44	Alb-pr2-F	GTATATTAGAGCGAGTCTTTC	Genotyping
45	cre2_R	ACCTCATCACTCGTTGCATC	Genotyping

Supplementary Table 2: Antibodies

Antigen	Species	Source	Cat. No.	Lot No.	Dil. (WB)	Immunofluorescence dilution, Fixation		
						Cardiomyocytes	Hepatocytes	HeLa
Na+/K+ ATPase alpha1 subunit	Mouse	Santa Cruz Biotehnology, inc.	SC-21712	G3004	1:1000			
GAPDH	Mouse	Biotrend Chemikalien	5G4	13/06-G4-C5	1:120000			
WRB	Rabbit	Synaptic systems	324 002		1:500			
CAML (Mouse cells)	Guinea pig	Synaptic systems	359 004		1:500			
TRC40 (Asna1)	Rabbit	Proteintech	15450-1-AP		1:1000			
Stx5	Rabbit	Synaptic systems	110053	110053/13	1:2000	1:200, PFA	1:100, MeOH	1:300, FA-Suc
Stx6	Rabbit	Synaptic systems	110062		1:1000	1:200, PFA		
Stx8	Rabbit	Synaptic systems	110083	110083/9	1:1000	1:200, PFA	1:300, MeOH	1:300, FA-Suc
Emerin	Rabbit	Santa Cruz Biotehnology, inc.	sc-15378	D2412	1:1000	1:150, MeOH		
Sec61β	Rabbit	Bernhard Dobberstein, Heidelberg			1:500	1:300, MeOH		
LBR	Rabbit	Proteintech	12398-1-AP		1:1000	1:500, PFA		
STT3B	Rabbit	Stephen High, Manchester			1:1000			
GM130	Mouse	BD Transduction Laboratories	610823	24277			1:200, MeOH	1:300, FA-Suc
p115	Mouse	BD Transduction Laboratories	612260			1:400, PFA		
Opsin	Mouse	Bernhard Dobberstein, Heidelberg			1:1000			
Poly-ubiquitin conjugate	Mouse	ENZO	BML-PW8810	5021240	1:1000			
LC3B	Rabbit	Cell Signaling	3868	0009	1:1000			
GFP	Mouse	Santa Cruz Biotehnology, inc.	sc-9996	F0713	1:1000			
FAM134B	Rabbit	Ivan Dikic, Frankfurt			1:1000			
p62/SQSTM1	Mouse	abcam	ab56416	GR282602-1	1:5000			
Sed5	Rabbit	Hans Dieter Schmidt, Göttingen			1:5000			
Vma2	Mouse	Molecular probes	A-6427		1:1000			

Supplementary Figure Legends

Supplementary Figure 1 Quantitative analysis of TA protein mRNAs. mRNA levels of investigated TRC40-pathway substrates relative to GAPDH cardiomyocytes and hepatocytes were determined by qPCR. Bars represent average +/- s.e.m. Variations are not significant by the student's t test.

Supplementary Figure 2 Control experiments addressing putative effects of tamoxifen-induced MerCreMer translocation to the nucleus in a WRB non-floxed ($Wrb^{wt/wt}$) Cre+ control mouse line. (a) TRC40-pathway components of ventricular cardiomyocytes isolated from 8-week old MerCreMer-positive, tamoxifen-induced control animals were analyzed by Western blot using GAPDH as loading control. An asterisk marks a cross-reactive protein. (b) Quantification of blots as in a. Bars represent average +/- s.e.m. (n=5, *p-value<0.05). (c) TA proteins of ventricular cardiomyocytes isolated from the MerCreMer-positive control animals were analysed by western blot using GAPDH as loading control. (d) Quantification of blots as in c; bars represent average +/- s.e.m. (n=5). (e) Cardiomyocytes were subjected to indirect immunofluorescence staining of TA proteins and confocal images were acquired. LBR, a multi-spanning membrane protein, served as a negative control. Scale bar: 20 µm.

Supplementary Figure 3 Colocalization of SNARE proteins with a Golgi marker. Cardiomyocytes isolated from $WRB^{fl/fl}$, Cre- and $WRB^{fl/fl}$, Cre+ littermates were subjected to indirect immunofluorescence for Stx5, Stx6 and Stx8 in combination with the Golgi marker protein p115. Images were acquired using a confocal microscope. Scale bar: 20 µm.

Supplementary Figure 4 Characterization of the consequences of WRB knockout in the liver. (a) Paraffin sections of liver were subjected to Goldner or HE staining. Representative images of a $WRB^{fl/fl}$, Cre- and a $WRB^{fl/fl}$, Cre+ 8-week old animal are shown. Original magnifications: images with Goldner staining 400x, images with HE staining 200x. Goldner staining was used to

reveal connective tissue. Nuclei appear in dark purple, cytosol in pink, and collagen in light blue. Hematoxylin and Eosin (HE) staining was used to assess general morphology. Nuclei are stained in dark purple, cytosol is stained in pink, and erythrocytes appear red. (b) Blood values of the liver enzymes Aspartate Transaminase (AST) and Alanine Transaminase (ALT) were measured in WRB$^{fl/fl}$ Cre- and Cre+ six-week old littermates. Graphs show average -/+ s.e.m. (n=9 (Cre-), n=7 (Cre+) ***p-value<0.001). (c) Cholesterol and triglyceride concentration in plasma of WRB$^{fl/fl}$ Cre- and Cre+ six-week old littermates. Graphs show average -/+ s.e.m. (n=4, the variations are not significant by student's t test). (d) Western blots assessing the correlation between residual WRB mRNA levels and the steady-state protein levels of different TRC40 pathway components in hepatocytes isolated from six-week old animals. Blots of Cre+ littermates of the 25th (++) and 50th (+) percentile of the boxplot in Fig. 2b, together with two Cre- animals are shown. Protein amounts were quantified relative to GAPDH and shown as a bar diagram for each individual blot. An asterisk marks a cross-reactive protein. (e) Western blots assessing the correlation between residual WRB mRNA levels and the steady-state protein levels of the TA proteins Stx5 and Stx8 in hepatocytes isolated from six-week old animals. The same samples as in c were used and analysis was performed as described for c.

Supplementary Figure 5 Steady-state protein levels and localization of TA proteins in *get* mutants lacking either the GET receptor, Get3, or both. (a) Live-cell microscopy images showing localization of selected genomically N-terminally GFP-tagged TA proteins in *S. cerevisiae get1/get2*, *get3*, and *get1/get2/get3* mutant strains. (b) Yeast strains depicted in panel (a) were analyzed by flow cytometry and fluorescence intensity distribution in wild type and *get* mutant strains are shown. BY4741 strain (gray trace) not expressing GFP was used as a negative control. (c) List of *S. cerevisiae* homologs of predicted human TA proteins indicating the localization of their genomically N-terminally GFP-tagged versions in wt and *get1/get2*, *get3*, and *get1/get2/get3* yeast strains based on live-cell microscopy. (d) Protein levels of native Sed5 were tested by western blot in wt, *get1/get2*, *get3*, and *get1/get2/get3* yeast

strains. The protein Vma2 was used as a loading control. (e) Quantification of blots from c. Bars represent average -/+ s.e.m. (n=3, **p-value<0.01).

Supplementary Figure 6 Comparison of Stx5 and Stx8 reveals different requirements for their cellular degradation. (a) Control siRNA treated HeLa cells or siRNA-mediated knockdown cells for WRB or TRC40 were incubated for 4 hours with 10 μM oprozomib (OPZ), an inhibitor of the proteasome, or DMSO as control. Cellular lysates were analyzed by Western blot for the indicated proteins. Graph in (b) shows quantification of the steady-state levels of Stx5 and Stx8 relative to the Na/K-ATPase. Bars represent average +/- s.e.m. (n=3) (c) Recombinant MBP-Stx5cyt and MBP-Stx8cyt were incubated at the indicated temperature. Soluble and insoluble proteins were separated by centrifugation. Fractions were loaded onto an SDS-PAGE gel and visualized by Coomassie staining. (d) Control silenced HeLa cells or knockdown cells for WRB or TRC40 were treated for 6 hours in the presence or absence of 100 μM chloroquine (CQ). Cellular lysates were analyzed by western blot for the indicated proteins. Graphs in (e-g) show quantification of the steady-state levels of TRC40, Stx5, and Stx8. Bars represent average +/- s.e.m. (n=6, *p-value<0.05, **p-value<0.01).

Supplementary Figure 1

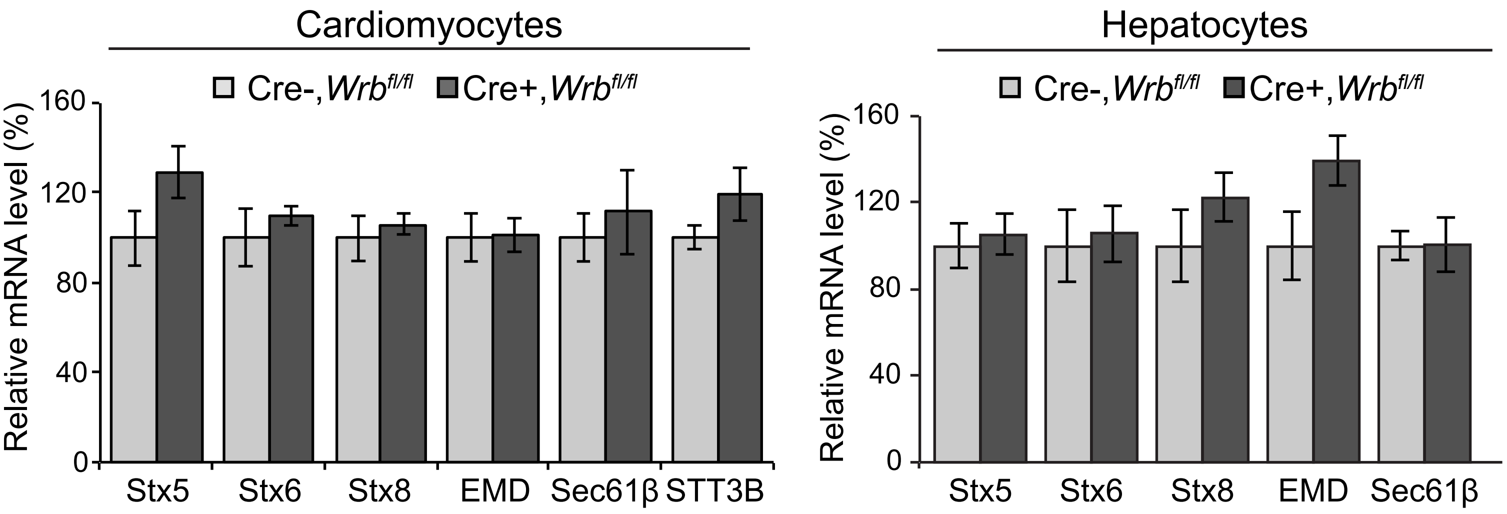

Supplementary Figure 2

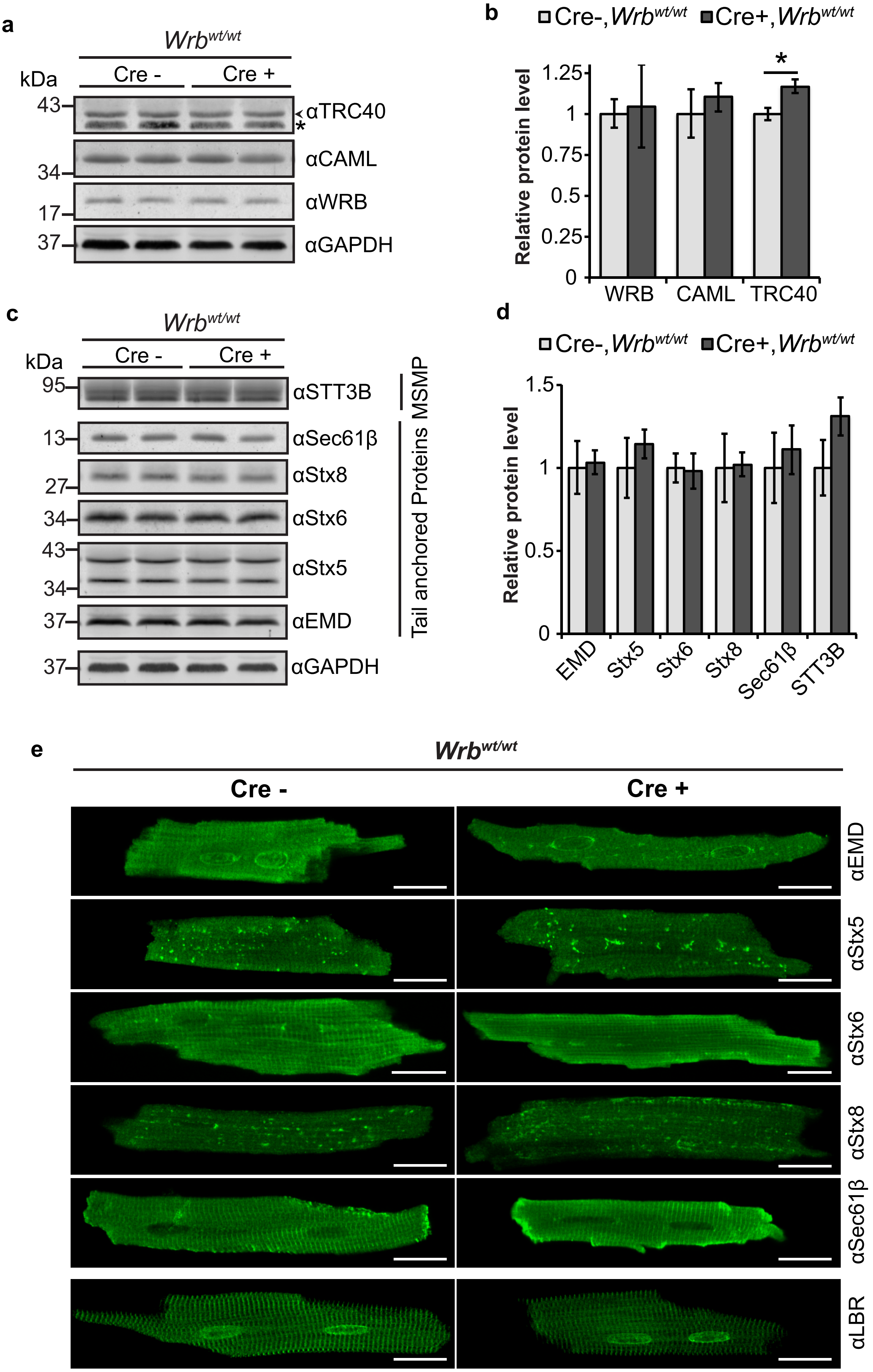

Supplementary Figure 3

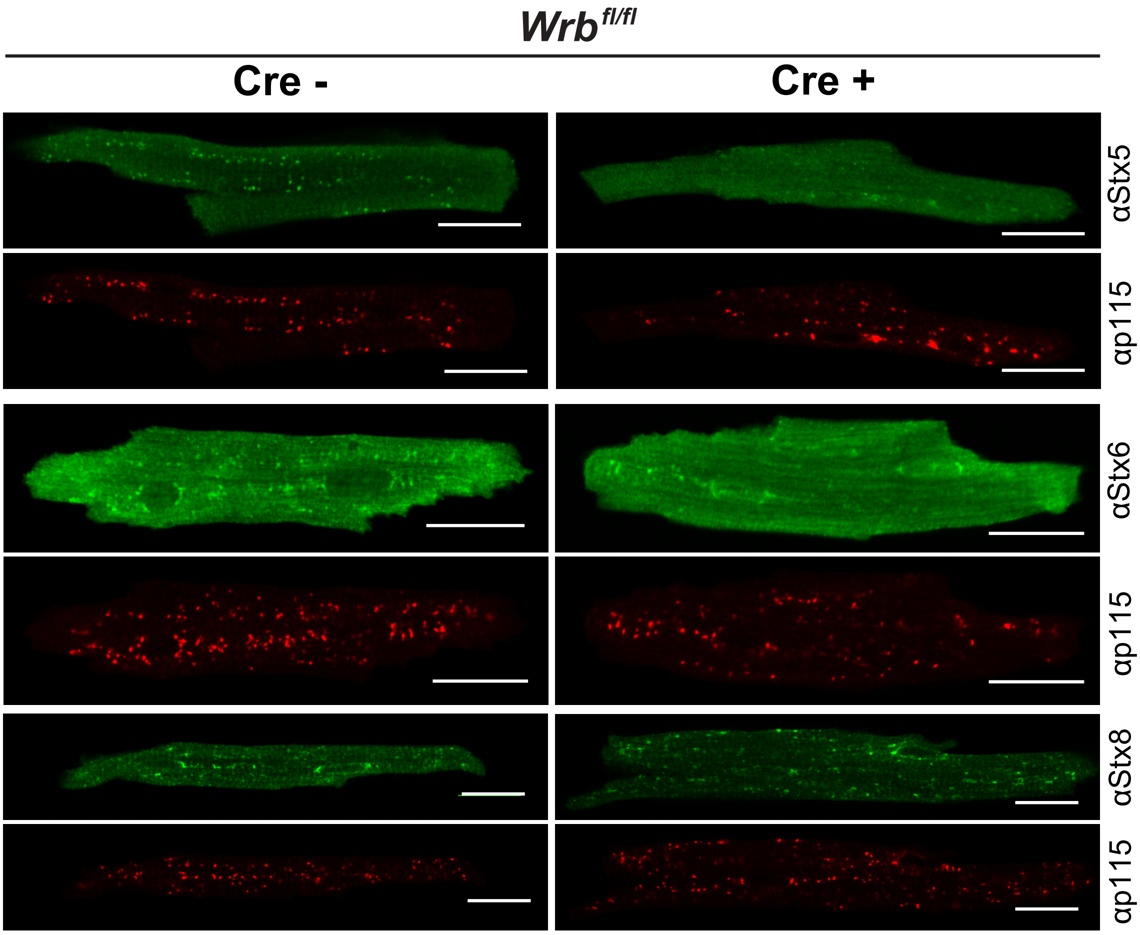

Supplementary Figure 4

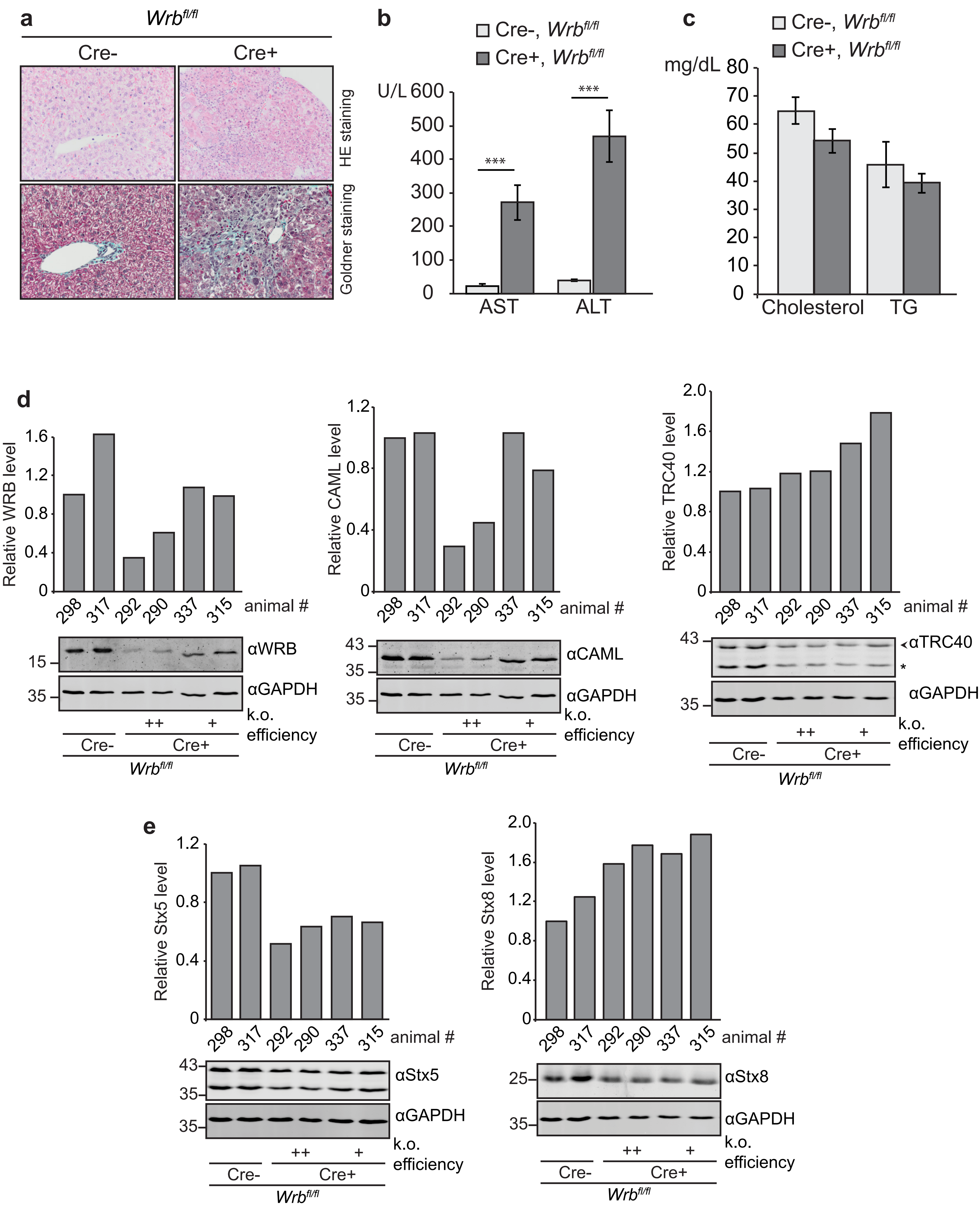

Supplementary Figure 5

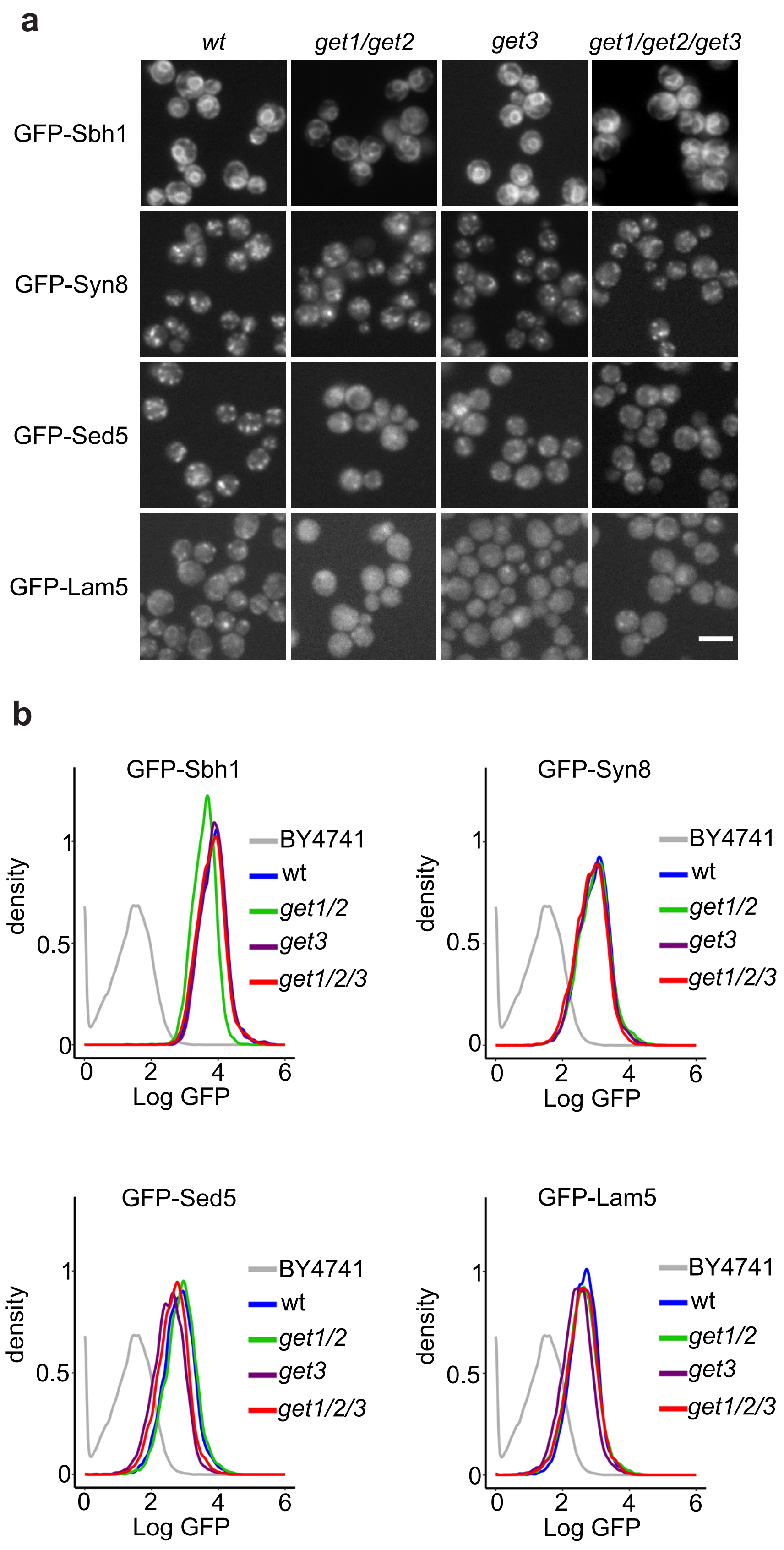

c

Gene	Localization in SWAT library	Change in localization in *get1/get2*, *get3*, *get1/get2/get3* strains
LAM5	punctate, ER	partial loss of puncta and ER staining
FMP32	faint	mitochondria
SED5	punctate	partial loss of puncta
BET1	punctate	no visible change
BOS1	punctate	no visible change
CYB5	ER	no visible change
FIS1	mitochondria	no visible change
GEM1	mitochondria	no visible change
GOS1	punctate	no visible change
HFD1	ER	no visible change
LAM6	punctate	no visible change
NYV1	vacuole	no visible change
PEP12	punctate	no visible change
SBH1	ER	no visible change
SCS2	ER	no visible change
SCS22	cytosol	no visible change
SEC20	ER	no visible change
SEC22	ER	no visible change
SFT1	cytosol	no visible change
SNC1	punctate, cytosol	no visible change
SNC2	punctate	no visible change
SSO1	vacuole	no visible change
SSO2	cell periphery	no visible change
SSS1	ER	no visible change
SYN8	punctate	no visible change
TLG1	punctate	no visible change
TLG2	punctate	no visible change
TOM7	mitochondria	no visible change
UBC6	ER	no visible change
USE1	ER	no visible change
VAM3	vacuole	no visible change
VPS64	ER	no visible change
VTI1	punctate	no visible change
YDL012C	bud tip, bud neck	no visible change
YDR034W-B	cell periphery	no visible change
YLR283W	ER	no visible change
YSY6	ER	no visible change
FAR10	faint	no visible change
UFE1	faint	no visible change
YSP2	faint	no visible change
COX16	not in library	not in library
HMX1	not in library	not in library
LAM4	not in library	not in library
SBH2	not in library	not in library
YDR210W	not in library	not in library

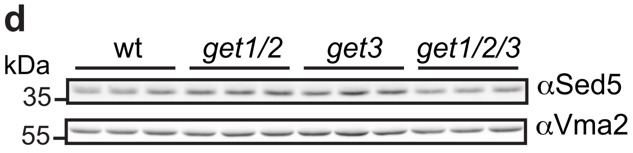

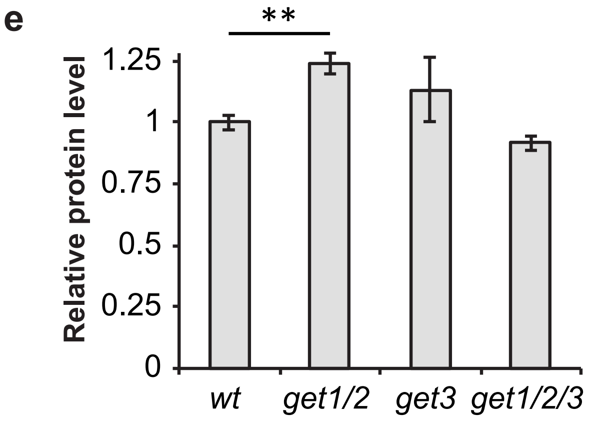

Supplementary Figure 6

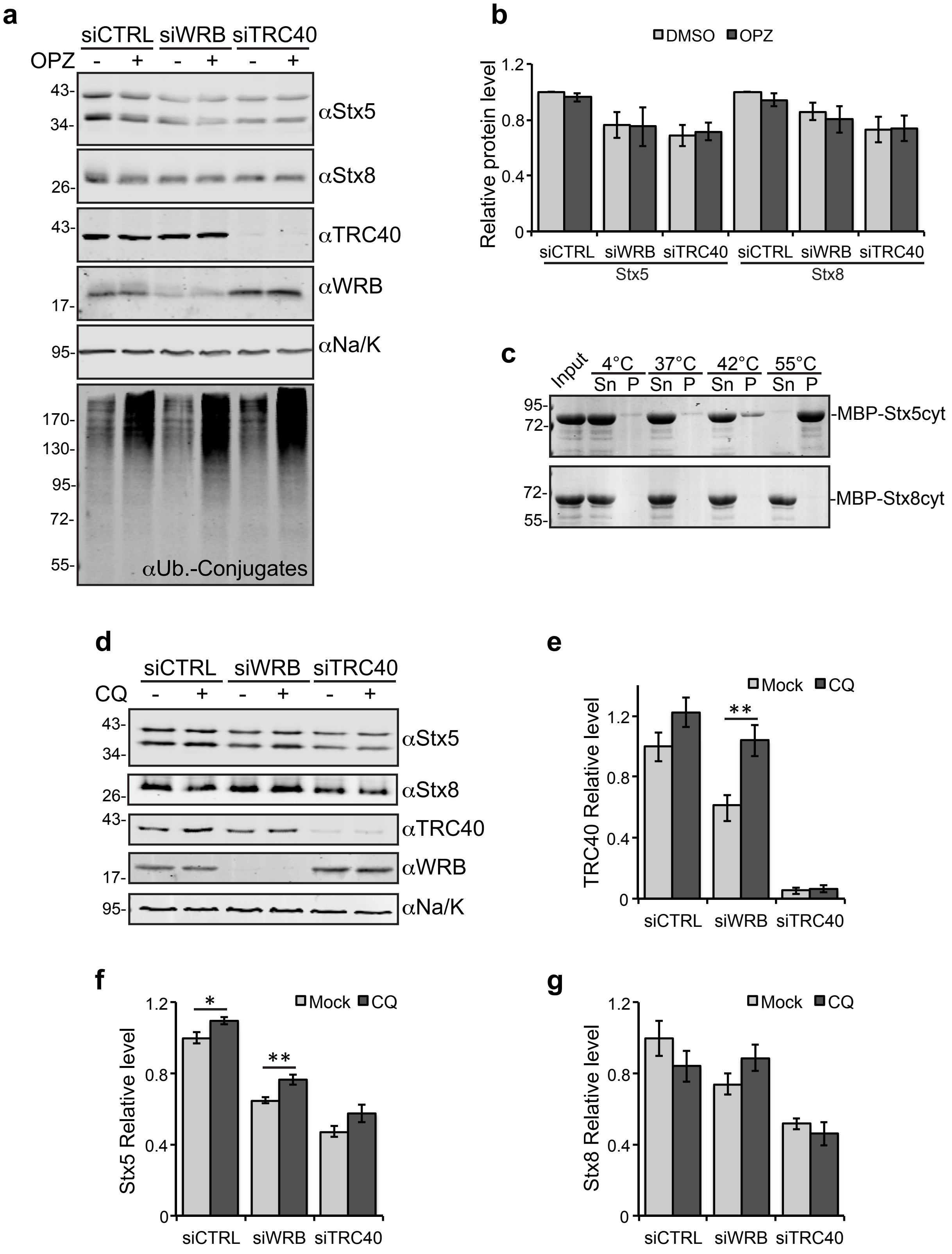

Supplement: Supplementary Figures [file srep39464-s1.pdf]
